# Direct Molecular Evidence for Desolvation-Controlled Lithium-Ion Insertion at Graphite Electrodes in Highly Concentrated Electrolytes

**DOI:** 10.1021/acs.jpclett.5c02274

**Published:** 2025-08-31

**Authors:** Saki Sawayama, Masaru Matsugami, Kenta Fujii

**Affiliations:** † Graduate School of Sciences and Technology for Innovation, 13150 Yamaguchi University, 1-16-2 Tokiwadai, Ube, Yamaguchi 755-8611, Japan; ‡ Faculty of Liberal Studies, National Institute of Technology, Kumamoto College, 2659-2 Suya, Koshi, Kumamoto 861-1102, Japan

## Abstract

Understanding the rate-determining step of lithium (Li)-ion insertion at graphite electrodes is essential for designing fast-charging Li-ion battery electrolyte systems. In this study, we quantitatively investigate how Li-ion solvation affects electrode reaction kinetics in highly concentrated electrolytes. By measuring the activation energy (*E*_a_) for the Li-ion insertion reaction in a series of 3.0 M LiFSA/solvent solutions, we found that *E*_a_ exhibited a strong linear correlation with the calculated binding energy (Δ*E*_bind_) of Li^+^–solvent interactions. This result provides direct evidence that, in highly concentrated electrolytes where Li^+^ is coordinated by both solvent molecules and anions to form ion-ordered structures, the desolvation of solvent molecules, rather than anion decoordination, controls the reaction kinetics. All-atom molecular dynamics (MD) simulations further revealed that, upon electrode polarization, FSA^–^ anions are preferentially excluded from the interfacial electrolyte structure closest to the electrode surface due to electrostatic repulsion, thereby inducing structural relaxation of the Li^+^ coordination shell. This yields a locally enriched environment of Li^+^ and solvent molecules, in which the disruption of Li^+^–solvent interactions (i.e., desolvation), rather than Li^+^–FSA^–^ interactions, controls the reaction rate and thus determines the activation energy.

## Graphical abstract

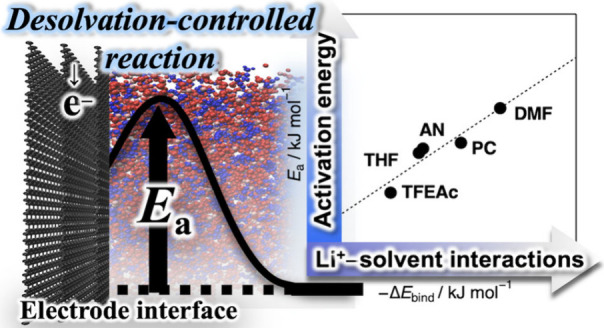

Rechargeable lithium-ion batteries (LIBs) have become the dominant power source for a wide range of portable electronic devices and electric vehicles due to their high energy density and excellent cycle life.^1^ As fast-charging capabilities become increasingly important, the interfacial kinetics of lithium-ion (Li^+^) insertion at graphite electrodes has attracted significant attention as a potential limiting factor for charging performance.^2^ In particular, understanding the rate-determining step of the Li^+^ insertion process is essential for optimizing electrolyte design and improving overall device performance.^3^

It has been established that in conventional carbonate-based electrolytes using cyclic and linear carbonates [e.g., ethylene carbonate (EC) and diethyl carbonate (DMC)], the activation energy (*E*_a_) for the Li^+^ insertion reaction is approximately 58 kJ mol^–1^, and the value is largely independent of the electrode material.^3–6^ This indicates that the interfacial kinetics are not controlled by Li^+^ diffusion within the solid phase, but rather by the solvation environment of Li^+^ in the electrolyte phase.^3,6,7^ However, in typical dilute electrolytes, investigating the influence of solvent–Li^+^ interactions on electrode kinetics is challenging because the electrochemical window is limited by the reductive instability of many solvents. This challenge originates from the fact that Li^+^ insertion occurs near the Li^+^/Li redox potential (−3.04 V vs SHE), placing the graphite electrode at a highly reducing potential where many organic solvents undergo electrochemical decomposition.^8^ As a result, most studies to date have focused only on a narrow range of stable carbonate solvents.^2,9,10^

Recently, highly concentrated electrolytes have emerged as a promising platform for both fundamental studies and battery applications.^11–13^ One of their unique features is the enhanced reductive stability, which is attributed to the formation of anion-derived solid electrolyte interphases (SEI).^14,15^ This stability enables the use of a wide variety of solvents, including those that are otherwise electrochemically unstable, thereby greatly enhancing the flexibility of electrolyte design.

In this study, we investigated how Li^+^ insertion kinetics at graphite electrodes are influenced by solvent species in highly concentrated electrolytes composed of LiFSA salt and various solvents [FSA: bis­(fluorosulfonyl)­amide]. First, we quantified the correlation between the experimentally determined *E*_a_ and the Li^+^–solvent interaction strength using experimentally and theoretically accessible indices. Furthermore, to obtain molecular-level insight into the desolvation process and interfacial structure, we performed all-atom molecular dynamics (MD) simulations under electrode polarization conditions.

To quantify Li^+^–solvent interactions, the Gutmann donor number (*D*_N_) is often used as a qualitative index of the solvent’s coordination strength with metal cations. *D*_N_ is defined as the molar enthalpy of reaction (−Δ*H*, in kcal mol^–1^) between a solvent and SbCl_5_ to form a 1:1 octahedral complex [SbCl_5_(solvent)] in a dilute 1,2-dichloroethane solution.^16^ However, *D*_N_ values are only available for a limited set of conventional solvents, and are not reported for many of the novel electrolyte solvents used in modern high-performance lithium-ion batteries. To address this limitation, we performed DFT calculations to evaluate the binding energy (Δ*E*_bind_) of Li^+^–solvent (1:1) complexes for 13 different organic solvents (Figure 1). The calculated −Δ*E*_bind_ values showed a strong linear correlation with the experimental *D*_N_ values:^16^ −Δ*E*_bind_ = 3.43 × *D*_N_ + 137. This finding confirms that −Δ*E*_bind_ can serve as a reliable quantitative indicator of Li^+^–solvent interaction strength, comparable to *D*_N_. Moreover, it enables estimation of Li^+^ coordination strength for novel solvents lacking *D*_N_ data.

**Figure 1:**
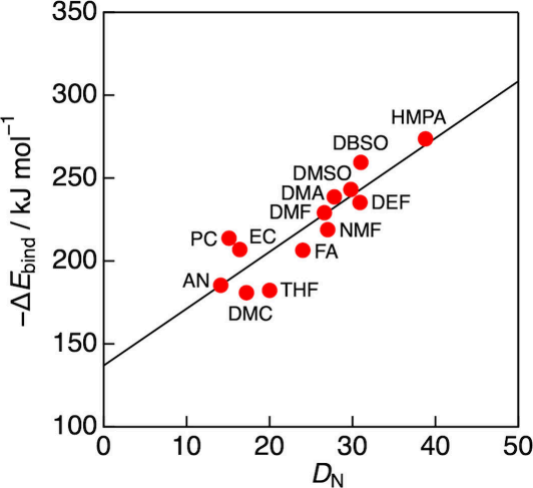
Calculated binding energies (Δ*E*_bind_) of Li^+^-solvent (1:1) complexes plotted against the experimental Gutmann’s donor numbers (*D*_N_) for 13 organic solvents; AN: acetonitrile, EC: ethylene carbonate, DMC: dimethyl carbonate, PC: propylene carbonate, THF: tetrahydrofuran, FA: formamide, NMF: *N*-methylformamide, DMF: *N,N*-dimethylformamide, DMA: *N,N*-dimethylacetamide, DMSO: dimethyl sulfoxide, DEF: *N,N*-dimethylformamide, DBSO: dibutyl sulfoxide, HMPA: hexamethylphosphoric triamide.

Figure 2 shows cyclic voltammograms (CVs) for the graphite electrode in 3.0 M LiFSA solutions prepared with six representative organic solvents (each chemical structure is shown in Figure S1), arranged in order of increasing Li^+^–solvent interaction strength: (a) TFEAc < (b) THF < (c) AN < (d) PC < (e) DMF < (f) HMPA. In the CV measurements, ethylene sulfite (ES) was used as an additive to promote the formation of a solid electrolyte interphase (SEI) on the graphite electrode. ES was added to the LiFSA solutions at a mole fraction of *x*_ES_ = 0.1 relative to the solvent+ES mixture (corresponding to 5–12 wt % ES, depending on the solvent). For solvents (a)–(e), clear redox currents corresponding to Li^+^ insertion and deinsertion were observed in the potential range of 0.0–0.8 V vs Li/Li^+^, although the insertion current was relatively small in the DMF system. In contrast, no redox current was detected for solvent (f), HMPA, suggesting that Li^+^ insertion is significantly suppressed. This suppression is attributed to the formation of a highly stable Li^+^–HMPA complex, reflecting the strong coordinating ability of HMPA due to its high electron-pair donating nature (−Δ*E*_bind_ = 273.7 kJ mol^–1^; *D*_N_ = 39.9). A distinct reductive peak appeared at ∼1.8 V during the first cathodic scan and disappeared in subsequent cycles. This behavior is attributed to the reductive decomposition of ES, leading to the formation of an ES-derived SEI layer on the graphite electrode.^17–19^ The presence of this SEI effectively suppresses the further reduction of organic solvents, which typically decompose at ∼0.5 V for TFEAc,^20,21^ ∼0.3 V for THF,^22^ 0.5–1.0 V for AN,^23–25^ and ∼0.5 V for DMF.^26^ However, no reductive peak near 1.8 V was observed in the HMPA system. We expect that this is due to the exclusion of ES molecules from the Li^+^ solvation shell in HMPA solutions. Raman spectra (Figure S2) of the LiFSA/HMPA+ES solution revealed that Li^+^ is coordinated exclusively by HMPA, with no detectable coordination by ES. This suggests that ES molecules are unlikely to be present near the graphite electrode when Li^+^–solvent complexes approach the surface during cathodic polarization.

**Figure 2:**
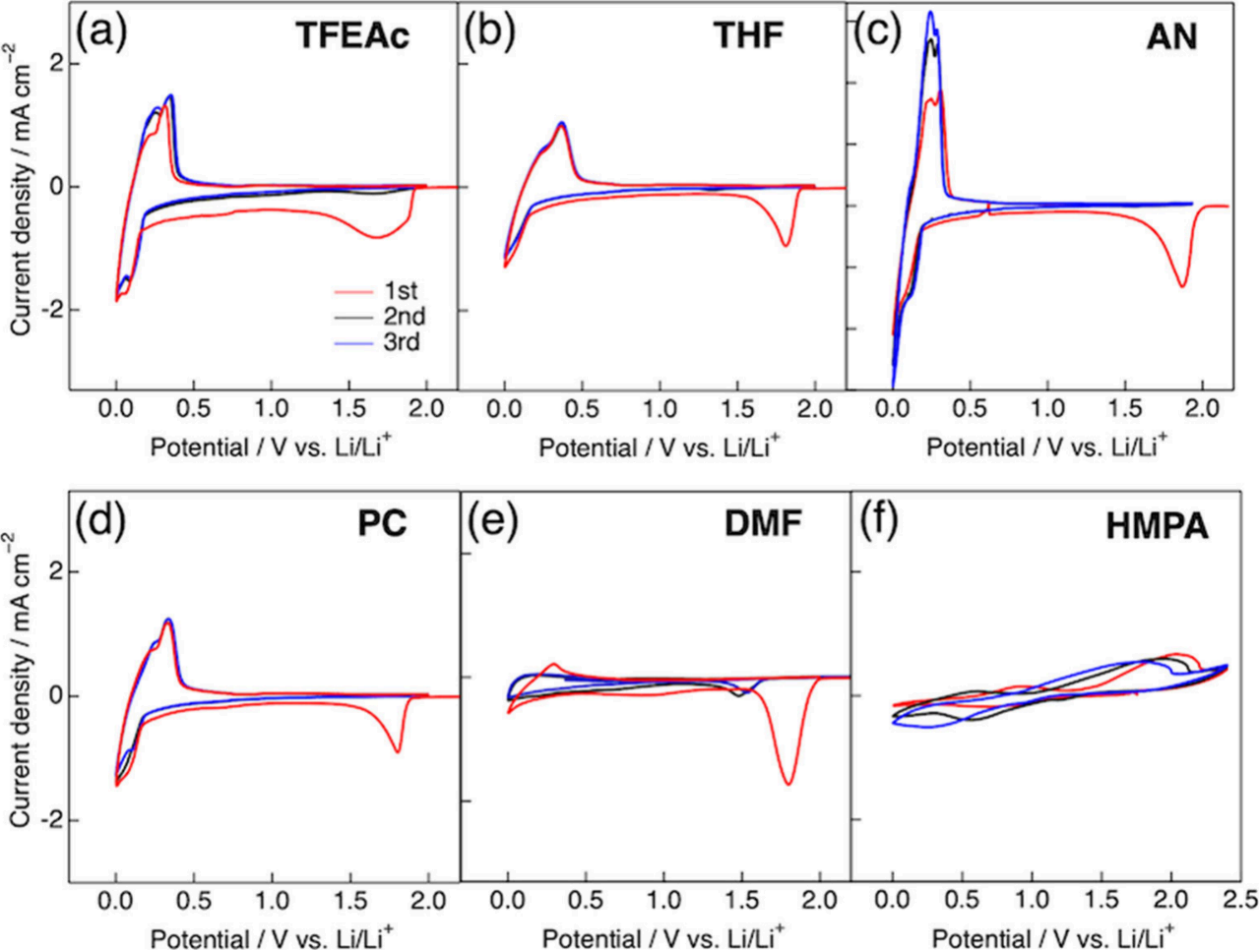
Cyclic voltammograms (CV) of a graphite electrode in 3.0 M LiFSA solutions containing ethylene sulfite (ES; *x*_ES_ = 0.1) with six representative organic solvents: (a) TFEAc, (b) THF, (c) AN, (d) PC, (e) DMF, and (f) HMPA. Scan rate: 0.2 mV s^–1^.

To determine the activation energy (*E*_a_) for the graphite anode reaction, we measured the AC impedance spectra for the LiFSA/X+ES systems (X: TFEAc, THF, AN, PC, and DMF) at various temperatures. From these spectra, we extracted the charge transfer resistance (*R*_ct_), i.e., Li^+^ transfer resistance at the electrode/electrolyte interface, as shown in Figure S3. In all systems, the impedance spectra measured at a fixed potential of 0.1 V vs Li/Li^+^ showed two semicircles: one in the high-frequency region and another in the low-frequency region. The diameters of both semicircles gradually decreased with increasing temperature. These spectra were analyzed using an equivalent circuit model commonly applied to Li^+^ insertion reactions in graphite electrodes, allowing the determination of *R*_ct_ values corresponding to the low-frequency semicircle at each temperature. Figure 3a shows the resulting *R*_ct_ values plotted as an Arrhenius plot based on the relation: 1/*R*_ct_ ∝ *A* exp­(−*E*_a_/*RT*),^5^ where *T*, *A*, and *R* are the temperature, pre-exponential factor, and gas constant, respectively. All systems exhibited linear Arrhenius behavior, enabling the extraction of *E*_a_ from the slopes. Importantly, the resulting *E*_a_ values showed a strong linear correlation with – Δ*E*_bind_, as shown in Figure 3b. This finding strongly suggests that weaker Li^+^–solvent interactions, i.e., energetically easier desolvation of Li^+^ complexes, lead to lower activation energies for the Li^+^ insertion reaction. To gain deeper insight into the reaction mechanism at the molecular level, we conducted all-atom molecular dynamics (MD) simulations, focusing particularly on the electrode/electrolyte interface in the highly concentrated electrolyte system, including structural rearrangements near the electrode surface and the Li^+^ solvation structure under electrochemical polarization.

**Figure 3:**
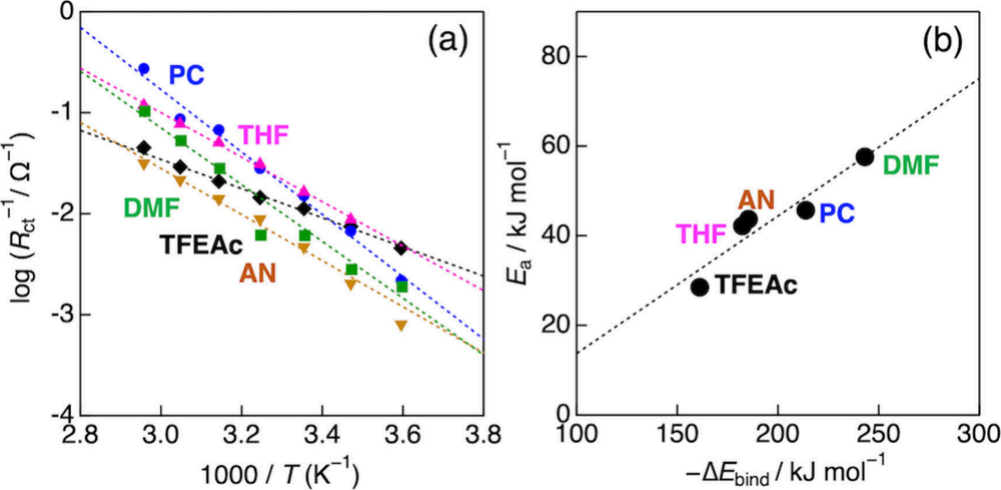
(a) Arrhenius plots of charge transfer resistance (*R*_ct_) for the graphite electrode in 3.0 M LiFSA solutions containing ethylene sulfite (ES; *x*_ES_ = 0.1), measured at a potential of 0.1 V vs Li/Li^+^. Solvents used: TFEAc (diamonds), THF (triangles), AN (inverted triangles), PC (circles), and DMF (squares). (b) Correlation between the activation energy (*E*_a_) for the Li^+^ insertion reaction and the calculated Li^+^-solvent binding energy (Δ*E*_bind_).

MD simulations were conducted using a slab model in which the electrolyte solution was confined between two graphene electrodes. Both the anode and cathode were modeled as planar graphene sheets. The dimensions of the simulation box were fixed in the X and Y directions, while the *Z*-axis length was adjusted to reproduce the experimental density of the electrolyte solution. Details of the simulation setup are provided in Table S2 in the Supporting Information. To simulate the effects of charged electrodes, partial charges (*q*) were assigned to the carbon atoms in the electrodes based on the fixed charge method (FCM).^27,28^ The resulting changes in interfacial structure as a function of electrode charge were then analyzed. Figure 4a presents the density profiles ρ­(*r*) of the individual components in the 3.0 M LiFSA/AN system, where Z = 0 Å corresponds to the anode surface. The density is plotted along the Z direction normal to the electrode up to *r* = 10 Å, and the full profile up to 130 Å (including the positive electrode side) is shown in Figure S4.

**Figure 4:**
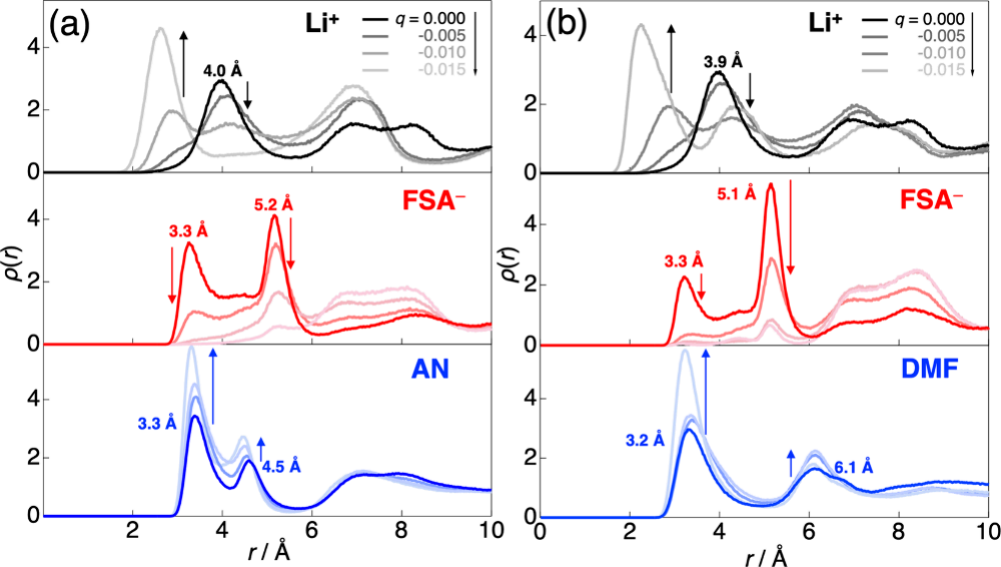
Density profiles ρ­(*r*) of Li^+^, FSA^–^, and solvent molecules along the *Z*-direction in 3.0 M LiFSA solutions with (a) AN and (b) DMF, obtained from MD simulations at various electrode charges (*q*). Z = 0 Å corresponds to the anode surface.

At *q* = 0 (no polarization), the first peaks appeared at 3.3 Å for both FSA^–^ and AN, with a slightly more distant peak at 4.0 Å for Li^+^. These peak positions reflect the molecular orientations near the electrode, as shown in Figure S5. The AN molecules were tilted such that the methyl groups pointed toward the electrode rather than adopting a vertical configuration that maximizes dipole stabilization. Consequently, the first peaks for C atoms (in AN) and Li^+^ (coordinated to AN) appeared at 3.3 Å and 4.0 Å, respectively. Similarly, the FSA anions were inclined with one SO_2_ group pointing toward the electrode, resulting in peaks at 3.3 Å (for the N atom in FSA) and 4.0 Å (for Li^+^). Beyond the first Li^+^ peak, the second layer was observed at 4.5 Å for AN and 5.2 Å for FSA^–^, followed by a broader Li^+^ peak at around 6.9 Å. As the *q* value increased (corresponding to progressive electrode polarization), these interfacial structures underwent significant changes. Here, the *q* values (0e ∼ – 0.015e) examined in the LiFSA/AN system correspond to interelectrode potential differences ranging from 0 to 4.15 V when accounting for charge fluctuations in the electrolyte near the polarized electrode surface (details are provided in the Supporting Information; Figure S6). Notably, the first and second peaks of FSA^–^ gradually diminished, while the first Li^+^ peak shifted closer to the electrode. In contrast, the AN peaks remained at the same positions, but their intensities increased monotonically. These *q*-dependent variations are clearly seen in the integrated ρ­(*r*) values shown in Figure S7. Similarly, the LiFSA/DMF system (Figure 4b) showed interfacial structuring analogous to that of the AN system. However, the *q*-dependent behavior of the ρ­(*r*) for FSA^–^ is particularly noteworthy: at higher *q* values, the first FSA^–^ peak is significantly reduced and nearly disappeared. To rationalize these interfacial structural changes observed during polarization, we propose a mechanistic model for the electrode reaction process during charging, as illustrated in Figure 5. In the bulk phase of highly concentrated electrolytes, Li ions are coordinated by both FSA anions and solvent molecules, forming ion aggregates in which multiple Li^+^ centers are connected via bridging FSA anions, as shown in our previous studies based on high-energy X-ray total scattering and MD simulations.^20,24,25,29,30^ This supposedly unique structure has in fact been commonly observed as a coordination motif in many highly concentrated electrolytes.^14,15,20,24,29,30^ We calculated the pair distribution functions of Li^+^ with respect to FSA^–^ and AN within the first interfacial layer (within 6 Å from the electrode surface), suggesting that similar coordination structures found in the bulk phase are also present near the electrode (Figure S8). That is, the interfacial electrolyte structure, in the absence of electrode polarization, largely reflects the bulk structure. As the electrode polarization increased (i.e., with increasing *q*), electrostatic repulsion between the negatively charged electrode and the FSA^–^ anions became more pronounced. As a result, the local concentration of FSA^–^ near the electrode surface decreased, resulting in the disruption of the ion-ordered structure (i.e., structural relaxation). In this process, FSA^–^ anions preferentially decoordinate from the Li^+^ coordination shell, while solvent molecules remain coordinated; this stage is designated as Step 1. In Step 2, Li^+^ ions migrate toward the electrode surface, resulting in an increased local concentration of Li^+^ near the interface. The electrochemical experiments described earlier demonstrated a strong linear correlation between the activation energy (*E*_a_) and the Li^+^–solvent binding energy (Δ*E*_bind_), indicating that the desolvation of solvent molecules, rather than the decoordination of FSA^–^ anions, is the rate-determining step in the Li^+^ insertion process. This rate-determining step is considered to occur at Step 3, where the local concentrations of Li^+^ and solvent molecules near the electrode surface are elevated due to the prior exclusion of FSA^–^. In this locally enriched environment, the disruption of Li^+^–solvent interactions (i.e., desolvation) is the key factor in controlling the activation energy of the reaction. This interpretation is further supported by considering an alternative scenario: if the rate-determining step were instead Step 2, namely, the initial removal of FSA^–^ from the Li^+^ coordination shell, the *E*_a_ values would correlate with the binding energy for Li^+^–FSA^–^ interactions rather than that for Li^+^–solvent interactions. Therefore, we conclude that under electrode polarization, the preferential decoordination of FSA^–^ from Li^+^ induces structural relaxation of the interfacial solvation shell, followed by desolvation of solvent molecules, after which solvent desolvation kinetically controls the Li^+^ insertion reaction at the graphite electrodes.

**Figure 5:**
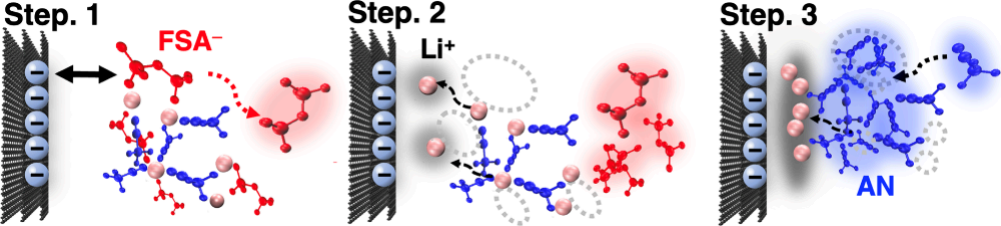
Schematic illustration of the proposed mechanism for Li^+^ insertion at graphite electrodes in highly concentrated electrolytes: Step 1, preferential decoordination of FSA^–^ due to electrostatic repulsion; Step 2, migration of Li^+^ toward the negatively charged electrode surface; Step 3, desolvation of solvent molecules, corresponding to the rate-determining step of the insertion reaction.

In summary, we demonstrated that the activation energy (*E*_a_) for Li^+^ insertion at graphite electrodes in highly concentrated electrolytes exhibits a strong linear correlation with the binding energy (−Δ*E*_bind_) of Li^+^–solvent interactions. This finding establishes that the strength of Li^+^–solvent interactions directly controls the reaction kinetics, highlighting desolvation as the rate-determining step. MD simulations further revealed that upon electrode polarization, FSA^–^ anions are preferentially excluded from the Li^+^ coordination shell near the electrode surface due to electrostatic repulsion, triggering a structural relaxation that facilitates subsequent desolvation of solvent molecules. These molecular-level insights elucidate the kinetic significance of interfacial solvation dynamics and provide guiding principles for the rational design of fast-charging electrolyte systems.

## Supplementary Material



## Notes

The Supporting Information is available free of charge on the ACS Publication Web site at DOI: The Supporting Information is available free of charge at https://pubs.acs.org/doi/10.1021/acs.jpclett.5c02274.

Experimental and computational methods, electrolyte compositions, parameters for MD simulations, chemical structures of solvents, Raman spectra, Nyquist plots, ρ­(*r*) profiles, orientation snapshots, interelectrode potential differences, integrated ρ­(*r*) values, and pair correlation functions *g*(*r*) (PDF)
