# Supplementary material for: Direct Molecular Evidence for Desolvation-Controlled Lithium-Ion Insertion at Graphite Electrodes in Highly Concentrated Electrolytes
Source: J Phys Chem Lett. 2025 Aug 31;16(36):9334–8. doi: 10.1021/acs.jpclett.5c02274 (PMC12434721; doi:10.1021/acs.jpclett.5c02274)
Supplement: Supplementary file 1 [file jz5c02274_si_001.pdf]

Supporting Information

Direct Molecular Evidence for Desolvation-Controlled Lithium-Ion Insertion at Graphite Electrodes in Highly Concentrated Electrolytes

Saki Sawayama,[a] *Masaru Matsugami,*[b] *and Kenta Fujii*[a]*

[a] Graduate School of Sciences and Technology for Innovation, Yamaguchi University, 1-16-2 Tokiwadai, Ube, Yamaguchi 755-8611, Japan

[b] Faculty of Liberal Studies, National Institute of Technology, Kumamoto College, 2659-2 Suya, Koshi, Kumamoto 861-1102, Japan

Corresponding Author

*E-mail: k-fujii@yamaguchi-u.ac.jp (K.F.)

Experimental and Computational Methods.

LiFSA salt (Kanto Chemical, battery grade) was vacuum-dried at 373 K for 24 h prior to use. TFEAc (2,2,2-trifluoroethyl acetate; TOSOH FINECHEM, battery grade), tetrahydrofuran (THF; FUJIFILM Wako Pure Chemical), acetonitrile (AN; FUJIFILM Wako Pure Chemical, super dehydrated), propylene carbonate (PC; Kishida Chemical), *N*,*N*-dimethylformamide (DMF; Kishida Chemical), and hexamethylphosphoric triamide (HMPA; Tokyo Chemical Industry Co.) were used without further purification. The chemical structures of each solvent are shown in Figure S1. Ethylene sulfite (ES; Sigma-Aldrich) was used as an additive after dehydration over 3 Å molecular sieves (FUJIFILM Wako Pure Chemical) for 24 h. The water content in the electrolyte solutions was confirmed to be below 100 ppm by Karl Fischer titration. The electrolyte solutions were prepared by weighing appropriate amounts of LiFSA salt and solvents at the desired molar ratio (Li salt:solvent) in an Ar-filled glovebox (O_2 and H_2O < 1 ppm), to prepare 3.0 M LiFSA/X + ES solutions (X = TFEAc, THF, AN, PC, DMF, and HMPA; mole fraction of ES in the X–ES mixture, x_{ES} = 0.1). The molar ratios, component concentrations (molarity /M), and experimental densities of the prepared electrolytes are listed in Table S1.

Cyclic voltammetry (CV) was carried out using a potentiostat (HZ-5000; Hokuto Denko) in a conventional three-electrode cell equipped with a graphite working electrode (1.6 mAh cm^{-2}, 10 mmϕ; Piotrek) and lithium foil as both counter (3.2 cm^2) and reference (1.0 cm^2) electrodes. The measurements were performed at a scan rate of 0.2 mV s^{-1}. Electrochemical AC impedance spectroscopy was performed using a potentio-galvanostat (SP-150; BioLogic) over a frequency range of 100 mHz to 1.0 MHz. The measurements were conducted in the same three-electrode cell at 0.1 V (Li/Li^+), under varying temperatures (278–338 K), to obtain impedance spectra. The spectra were analyzed using an equivalent circuit model commonly applied to graphite electrodes

for Li^+ insertion reactions to extract the charge-transfer resistance (R_{ct}). Activation energies (E_a) were calculated from the temperature dependence of R_{ct} using the Arrhenius equation: $1/R_{ct} \propto A\exp(-E_a/RT)$, where T, A, and R denote the temperature, pre-exponential factor, and gas constant, respectively. The slope in the $\log(1/R_{ct})$ vs. $1/T$ plot yields E_a. DFT calculations were carried out using Gaussian 09 software.[1] The geometries of Li^+–solvent (1:1) complexes were optimized at the B3LYP/6-311G** level, followed by frequency analysis to confirm local energy minima. Binding energy (ΔE_{bind}) was calculated as the SCF energy difference between the Li-solvent complex and its individual components (Li^+ and solvent): $\Delta E_{bind} = E_{SCF}(\text{complex}) - E_{SCF}(Li^+) - E_{SCF}(\text{solvent})$, and corrected for basis set superposition error using the counterpoise method[2].

All-atom molecular dynamics (MD) simulations were performed using GROMACS 2021.7 to investigate 3.0 M LiFSA/X (X = AN or DMF) electrolytes confined between two graphite electrodes. Both the anode and cathode were modeled as planar graphene sheets. The simulation box was fixed in the X and Y directions, while the Z-dimension was adjusted to reproduce the experimental density of each electrolyte solution. The electrolyte compositions and simulation box lengths are summarized in Table S2. Initial configurations were generated using the PACKMOL package[3]. Simulations were carried out in the *NVT* ensemble at 298 K with a 2 fs time step. To properly implement 2D periodic boundary conditions, slab corrections[4] were applied, and a vacuum layer equal to the Z-directional length in the simulation box was added on the electrode side not in contact with the electrolyte. First, a 15 ns equilibration was conducted with both electrodes set to zero net charge. Subsequently, partial charges (q) were assigned to the carbon atoms in the electrodes using the fixed charge method (FCM)[5-6]: q = –0.005e, –0.01e, and –0.015e for the negative electrode, with the corresponding positive charges assigned to the positive electrode. For each charge condition, an additional 5 ns equilibration was performed, followed by

a 30 ns production run. The trajectories from the production runs were used to calculate the density profiles $\rho(r)$, Poisson potential profiles[7] (Figure S6), and pair correlation functions $g(r)$ (Figure S8). The force fields employed in this MD simulation include the CLaP model for FSA^- and the OPLS-AA parameters for AN and DMF, encompassing both intermolecular interactions (i.e., Lennard–Jones and Coulombic terms) and intramolecular interactions (i.e., bond stretching, angle bending, and torsional rotations).[8-11] The Lennard–Jones parameters for Li^+ were adopted from a previous study of Li salt in carbonate-based solvents.[12] For AN, partial atomic charges (q) were determined by DFT calculations using the ChelpG method at the MP2/cc-pVTZ(-f)//HF/6-31G* level,[13] yielding values of –0.269 (C), +0.113 (H), +0.427 (C), and –0.497 (N) for the $C\text{-}H_3\text{-}C{\equiv}N$ moiety. The charges for DMF were adopted from literature values without modification.[11] In contrast, the partial charges of Li^+ and FSA^- were scaled from their original values to reflect the influence of strong ion–ion interactions in the highly concentrated electrolyte. In our previous study, we established that applying a scaling factor of $f = 0.6$ provides good agreement between simulated and experimental radial distribution functions,[9, 14] and this value was adopted in the present simulations.

Table S1. Electrolyte composition data for 3.0 M LiFSA/X+ES solutions with x_{ES} = 0.1 (X = TFEAc, THF, AN, PC, DMF, and HMPA): solvent species (X), molar ratio of salt to solvent (X:ES), concentrations of solvent X (c_X) and ES (c_{ES}), the added amount of ES (wt%), the experimental density (d), and viscosity (η) of each solution.

solvent (X)	LiFSA : X : ES	c_X / M	c_{ES} / M	ES / wt%	d / g cm^{-3}	η / mPa·s
TFEAc	1 : 2.2 : 0.25	6.54	0.74	5.12	1.490	14.16
THF	1 : 3.1 : 0.34	9.18	1.03	8.32	1.224	56.61
AN	1 : 4.7 : 0.52	14.01	1.56	12.9	1.142	2.58
PC	1 : 3.4 : 0.38	10.21	1.15	7.85	1.610	3.65
DMF	1 : 3.2 : 0.36	9.63	1.08	8.38	1.270	11.81
HMPA	1 : 6.7 : 0.74	5.32	0.59	5.55	1.102	5.34

Table S2. Parameters for the MD simulations of 3.0 M LiFSA/AN (upper) and LiFSA/DMF (lower): number of LiFSA ion pairs and solvent molecules, simulation box length (Å), and simulated density (d).

System	Li-FSA	Solvent (AN, DMF)	bulk box length / (x × y × z) / Å	d / g cm^{-3}	
				MD	Exp.[a]
3.0 M LiFSA/AN	1750	2100	61.8 × 60.3 × 135	1.133	1.133
3.0 M LiFSA/DMF	1970	2364	61.8 × 60.3 × 106	1.235	1.235

[a] Experimental values.

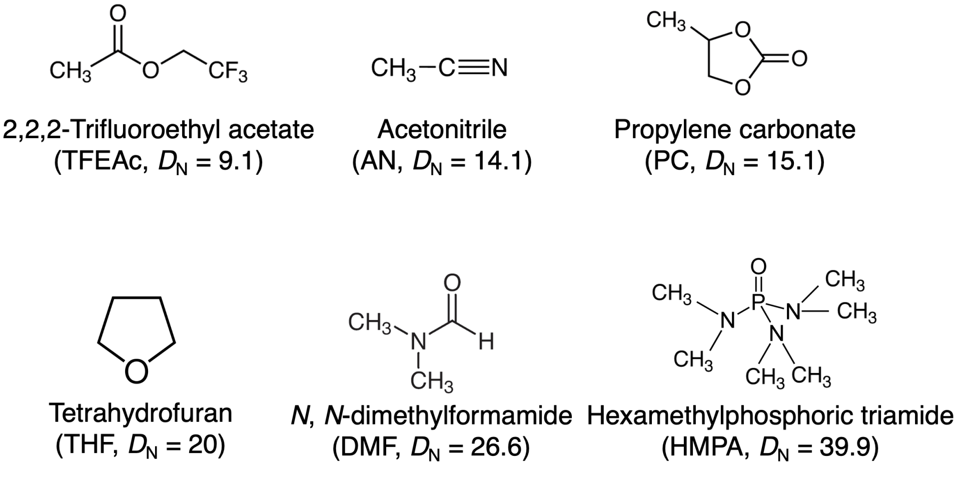

Figure S1. Chemical structures of the solvent molecules used in this study.

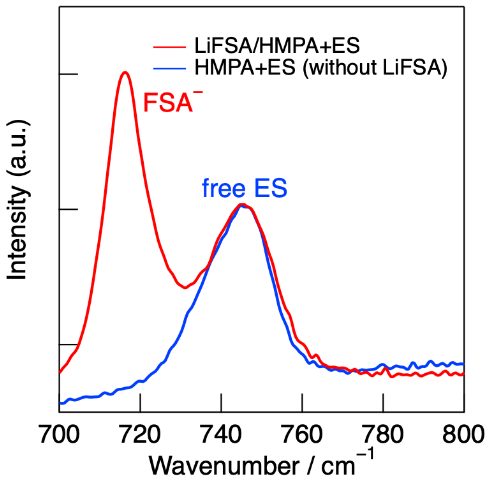

Figure S2. Raman spectra of 0.8 M LiFSA/HMPA+ES (red) and HMPA+ES (blue) solutions. The spectra were obtained using a 532 nm-laser with an optical resolution of 4.0 cm^{-1} in a quartz cell. Our previous work established that ethylene sulfite (ES) molecules coordinated to Li^+ exhibite a distinct peak shifted to higher frequency relative to that of free ES.[15] In the case of the LiFSA/HMPA+ES solution with x_{ES} = 0.1, no such bound ES peak observed—only the peak corresponding to free ES was present, similar to the spectrum of the HMPA+ES mixture without LiFSA. This absence indicates that ES molecules do not coordinate to Li^+ in the presence of strongly coordinating HMPA molecules.

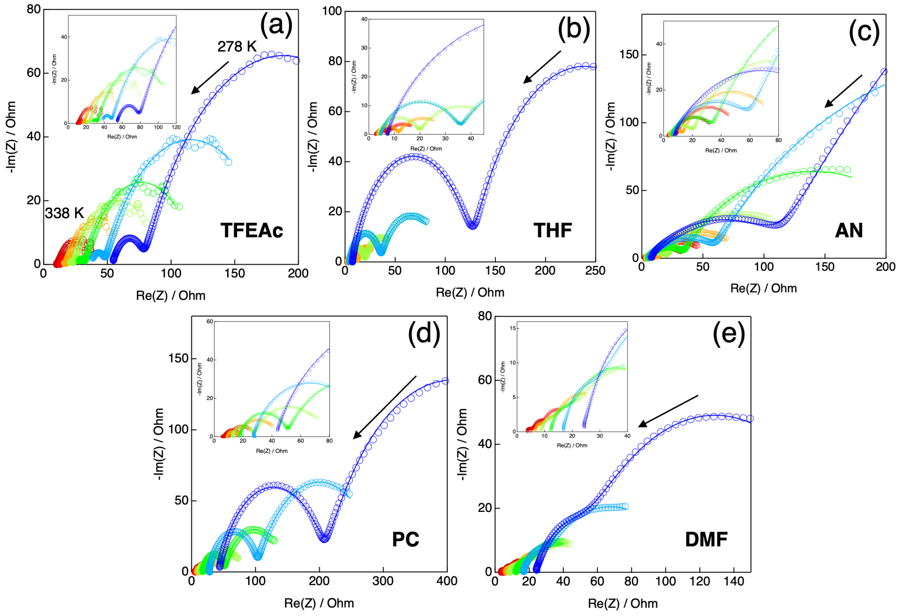

Figure S3. Temperature dependent Nyquist plots for the graphite electrode in 3.0 M LiFSA/X+ES solutions [X = (a) TFEAc, (b) THF, (c) AN, (d) PC, and (e) DMF] at a fixed potential of 0.1 V.

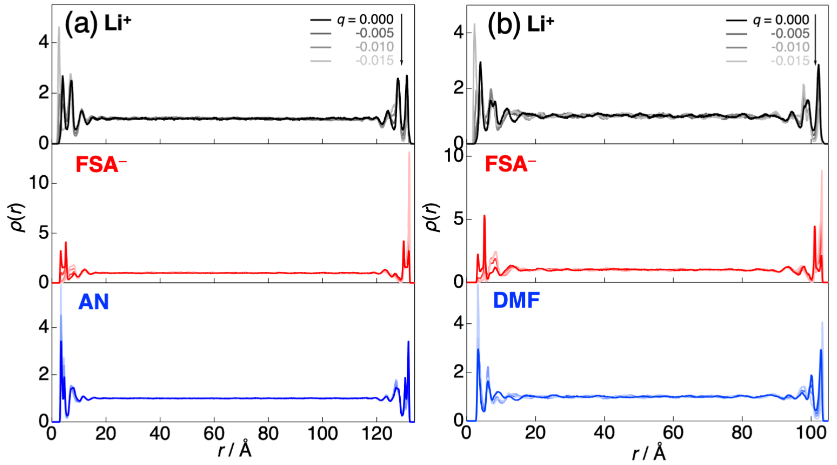

Figure S4. Density profiles $\rho(r)$ of Li^+, FSA^-, and solvent molecules along the whole Z-direction (from anode to cathode) in 3.0 M LiFSA solutions with (a) AN and (b) DMF, obtained from MD simulations at various electrode charges (q). The Z = 0 Å corresponds to the anode surface.

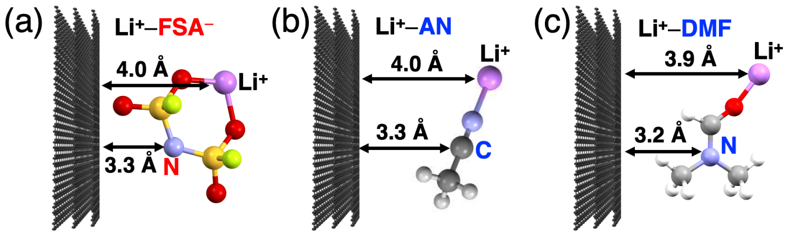

Figure S5. Orientations of (a) Li^+–FSA^-, (b) Li^+–AN, and (c) Li^+–DMF complexes relative to the anode surface.

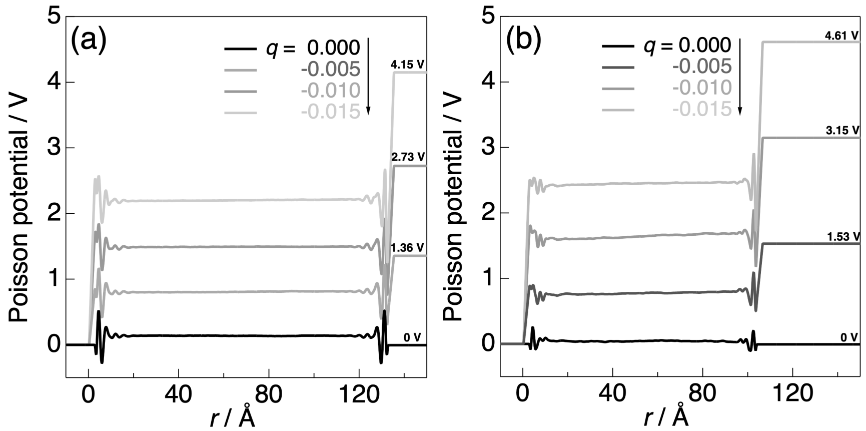

Figure S6. Poisson potential profiles as a function of q values in MD simulations for 3.0 M LiFSA solutions with (a) AN and (b) DMF.

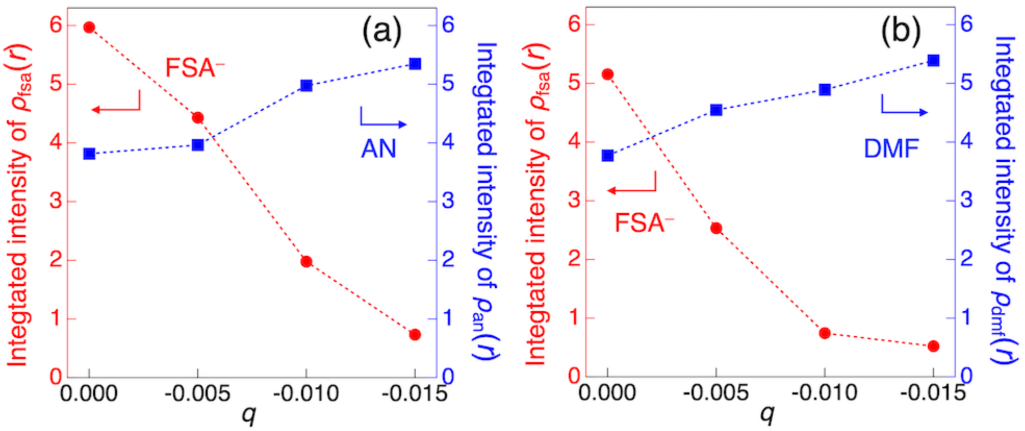

Figure S7. Integrated $\rho(r)$ values (up to 6 Å) for solvent molecules (blue squares) and FSA^{-} anions (red circles) in (a) 3.0 M LiFSA/AN and (b) 3.0 M LiFSA/DMF systems near the electrode interface.

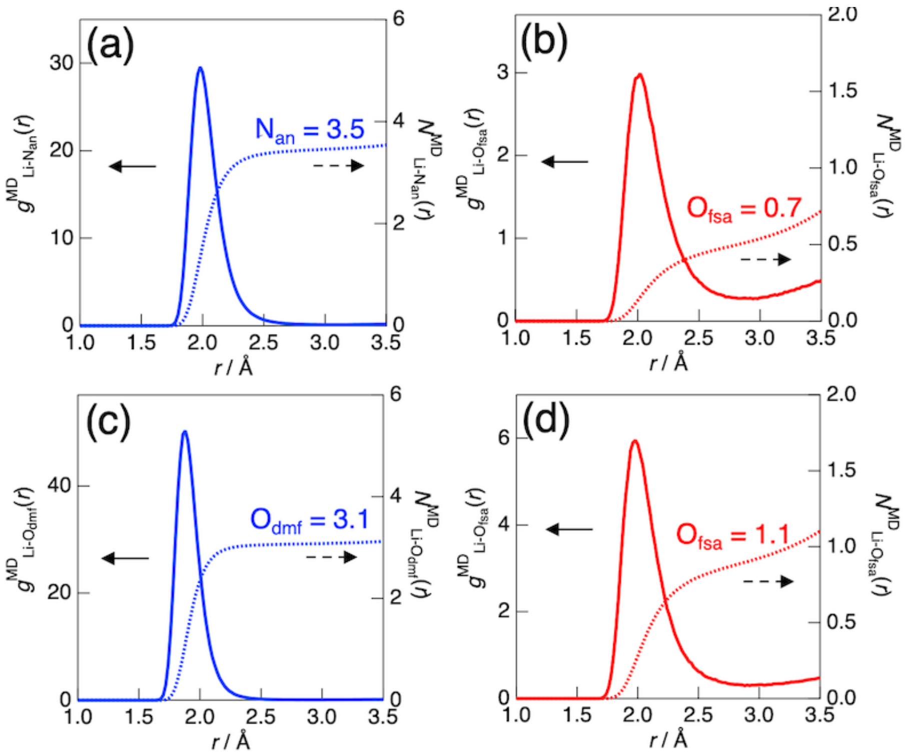

Figure S8. Atom–atom pair correlation functions ($g^{MD}_{Li\text{-}X}(r)$; left axis, solid lines) for target X atoms (N or O) of (a) AN, (b) FSA^{-} around Li^{+} near the anode interface (within 6 Å, $q = 0$) in the 3.0 M LiFSA/AN system, and (c) DMF and (d) FSA^{-} around Li^{+} in the 3.0 M LiFSA/DMF system, along with their integrated coordination number profiles $N(r)$ (right axis, dashed lines).

References

(1) Frisch, M. J.; Trucks, G. W.; Schlegel, H. B.; Scuseria, G. E.; Robb, M. A.; Cheeseman, J. R.; Scalmani, G.; Barone, V.; Mennucci, B.; Petersson, G. A. Gaussian 09; Gaussian, Inc. *Wallingford, CT* **2009,** *6492*.

(2) Asada, M.; Fujimori, T.; Fujii, K.; Kanzaki, R.; Umebayashi, Y.; Ishiguro, S. Solvation structure of magnesium, zinc, and alkaline earth metal ions in *N*,*N*-dimethylformamide, *N*,*N*-dimethylacetamide, and their mixtures studied by means of Raman spectroscopy and DFT calculations –Ionic size and electronic effects on steric congestion–. *J. Raman Spectrosc.* **2007,** *38*, 417-426.

(3) Martinez, L.; Andrade, R.; Birgin, E. G.; Martinez, J. M. PACKMOL: a package for building initial configurations for molecular dynamics simulations. *J. Comput. Chem.* **2009,** *30*, 2157-2164.

(4) Yeh, I.-C.; Berkowitz, M. L. Ewald summation for systems with slab geometry. *J. Chem. Phys.* **1999,** *111*, 3155-3162.

(5) Siepmann, J. I.; Sprik, M. Influence of surface topology and electrostatic potential on water/electrode systems. *J. Chem. Phys.* **1995,** *102*, 511-524.

(6) Feng, G.; Zhang, J. S.; Qiao, R. Microstructure and Capacitance of the Electrical Double Layers at the Interface of Ionic Liquids and Planar Electrodes. *J. Phys. Chem. C* **2009,** *113*, 4549–4559.

(7) Wang, Z.; Olmsted, D. L.; Asta, M.; Laird, B. B. Electric potential calculation in molecular simulation of electric double layer capacitors. *J. Phys. Condens.* **2016,** *28*, 464006.

(8) Shimizu, K.; Almantariotis, D.; Gomes, M. F. C.; Pádua, A. A. H.; Lopes, J. N. C. Molecular Force Field for Ionic Liquids V: Hydroxyethylimidazolium, Dimethoxy-2-Methylimidazolium, and Fluoroalkylimidazolium Cations and Bis(Fluorosulfonyl)Amide, Perfluoroalkanesulfonylamide, and Fluoroalkylfluorophosphate Anions. *J. Phys. Chem. B* **2010,** *114*, 3592–3600.

(9) Sawayama, S.; Morinaga, A.; Mimura, H.; Morita, M.; Katayama, Y.; Fujii, K. Fluorophosphate-Based Nonflammable Concentrated Electrolytes with a Designed Lithium-Ion-Ordered Structure: Relationship between the Bulk Electrolyte and Electrode Interface Structures. *ACS Appl. Mater. Interfaces* **2021,** *13*, 6201-6207.

(10) Price, M. L. P.; Ostrovsky, D.; Jorgensen, W. L. Gas-Phase and Liquid-State Properties of Esters, Nitriles, and Nitro Compounds with the OPLS-AA Force Field. *J. Comput. Chem.* **2001,** *22*, 1340–1352.

(11) Vasudevan, V.; Mushrif, S. H. Force field parameters for *N*,*N*-Dimethylformamide (DMF) revisited: Improved prediction of bulk properties and complete miscibility in water. *J. Mol. Liquids* **2015,** *206*, 338-342.

(12) Soetens, J.-C.; Millot, C.; Maigret, B. Molecular Dynamics Simulation of $Li^+BF_4^-$ in Ethylene Carbonate, Propylene Carbonate, and Dimethyl Carbonate Solvents. *J. Phys. Chem. A* **1998,** *102*, 1055-1061.

(13) Curt M. Breneman; Wiberg, K. B. Determining Atom-Centered Monopoles from Molecular Electrostatic Potentials. The Need for High Sampling Density in Formamide Conformational Analysis. *J. Comput. Chem.* **1990,** *11*, 361-373.

(14) Sogawa, M.; Sawayama, S.; Han, J.; Satou, C.; Ohara, K.; Matsugami, M.; Mimura, H.; Morita, M.; Fujii, K. Role of Solvent Size in Ordered Ionic Structure Formation in Concentrated Electrolytes for Lithium-Ion Batteries. *J. Phys. Chem. C* **2019,** *123*, 8699-8708.

(15) Suzuki, K.; Sawayama, S.; Deguchi, Y.; Sai, R.; Han, J.; Fujii, K. A structural and electrochemical study of lithium-ion battery electrolytes using an ethylene sulfite solvent: from dilute to concentrated solutions. *Phys. Chem. Chem. Phys.* **2022,** *24*, 27321-27327.
